# *"I don't eat a hamburger and large chips every day!" *A qualitative study of the impact of public health messages about obesity on obese adults

**DOI:** 10.1186/1471-2458-10-309

**Published:** 2010-06-04

**Authors:** Sophie Lewis, Samantha L Thomas, Jim Hyde, David Castle, R Warwick Blood, Paul A Komesaroff

**Affiliations:** 1 Consumer Health Research Group (CHaRGe) Primary Care Research Unit, School of Primary Health Care, Monash University, Building 1, 270 Ferntree Gully Rd, Notting Hill, Victoria 3168, Australia; 2 Victorian Department of Health, GPO Box 4047, Melbourne, Victoria 3001, Australia; 3 Department of Psychiatry, University of Melbourne, PO Box 2900, Fitzroy, Victoria 3065, Australia; 4 News Research Group, Faculty of Arts and Design, University of Canberra, Canberra, ACT 2601, Australia; 5 Centre for Ethics in Medicine and Society, Monash University, Department of Medicine, Monash Medical School, Alfred Hospital, Prahran, Victoria 3181, Australia

## Abstract

**Background:**

We are a society that is fixated on the health consequences of 'being fat'. Public health agencies play an important role in 'alerting' people about the risks that obesity poses both to individuals and to the broader society. Quantitative studies suggest people comprehend the physical health risks involved but underestimate their own risk because they do not recognise that they are obese.

**Methods:**

This qualitative study seeks to expand on existing research by exploring obese individuals' perceptions of public health messages about risk, how they apply these messages to themselves and how their personal and social contexts and experiences may influence these perceptions. The study uses in depth interviews with a community sample of 142 obese individuals. A constant comparative method was employed to analyse the data.

**Results:**

Personal and contextual factors influenced the ways in which individuals interpreted and applied public health messages, including their own health and wellbeing and perceptions of stigma. Individuals felt that messages were overly focused on the physical rather than emotional health consequences of obesity. Many described feeling stigmatised and blamed by the simplicity of messages and the lack of realistic solutions. Participants described the need for messages that convey the risks associated with obesity while minimising possible stigmatisation of obese individuals. This included ensuring that messages recognise the complexity of obesity and focus on encouraging healthy behaviours for individuals of all sizes.

**Conclusion:**

This study is the first step in exploring the ways in which we understand how public health messages about obesity resonate with obese individuals in Australia. However, much more research - both qualitative and quantitative - is needed to enhance understanding of the impact of obesity messages on individuals.

## Background

As a society we are more than ever aware of the perceived effects of 'being fat'. Public health agencies play a key role in 'alerting' individuals to the different risks associated with obesity and the urgent need for intervention and self-regulation. Research finding links between risk and obesity can be clustered into a number of categories. Firstly are the associations between obesity and physical health risks. These risks include Type II diabetes, cardiovascular disease, gallbladder disease, and some types of cancer such as breast, endometrial, colorectal and kidney cancers [1]. Secondly are the associations between poor emotional health and obesity. Whilst there is debate about whether obesity leads to poor mental health, or vice versa, obese individuals have higher rates of depression, mood or anxiety disorders and suicide than the general population [2-4]. Thirdly are the social consequences of obesity. Research has shown that obese individuals experience stigma and discrimination [5], reduced employment and educational opportunities [6] and social isolation [7]. This research - particularly around the physical health consequences of 'being fat' - has stimulated a variety of obesity prevention responses from public health agencies aimed at informing the general population about the health risks associated with obesity, encouraging individuals to avoid risky behaviours and promoting the benefits of preventive activities such as healthy eating and regular exercise.

Despite the vital importance of these public health activities there is only limited evidence to suggest that having a heightened perception of the health risks of obesity leads to increased intentions, efforts, and success in losing weight [8,9]. Furthermore we have limited knowledge of how obese individuals interpret and apply population based information about health risks to themselves and whether these messages have any positive or negative impacts on them such as increasing weight-based stigma. A small number of quantitative studies show that obese individuals underestimate their own health risks because they do not recognise that they are obese [10-12]. Some of these studies also suggest that socio-demographic factors strongly influence these interpretations [13-15]. However, we have limited in depth understanding of how socio-cultural and personal experiences of illness may interact with public health messages aimed at highlighting these risks.

Socio-cultural risk communication frameworks recognise that an individual's response to 'risk' is mediated by a myriad of personal experiences as well as the social context in which these messages are received [16,17]. A number of different factors may influence how individuals interpret and apply messages about health risk. The first is the influence of social, cultural, geographic and economic factors such as gender, age, occupation, nationality, sexual identity and power relationships [18-20]: for example socio-economic circumstances may limit people's access to fresh fruit and vegetables and recreational facilities [21]. Associated with this is the extent to which individuals feel that they are able to influence the health conditions that affect them [22]. If obese individuals perceive that there are few comprehensive, accessible, affordable and sustainable long term supports to help them to improve their health and wellbeing this may influence their sense of control or power over their health outcomes and the different levels of importance they place on different types of risk.

Trust also plays a role in the way individuals interpret and act upon messages. Individuals give more credibility to sources of information with which they are familiar - for example family, friends, or health professionals with whom they have formed a relationship - and may be sceptical about the validity of government messages about risk [23,24]. This is particularly true when an excess of risk messages conflict with people's personal experiences of their health and wellbeing [25].

Working across each of these factors is the role of stigma. Stigma may play a key role in influencing the importance individuals place on different types of risk; their attitudes towards these risks, the extent to which they trust certain sources of information and accept or seek treatment, and their engagement in high risk behaviours. There is no doubt that obesity is one of the most stigmatised health conditions in society today [6]. If obese individuals feel stigmatised by the messages they receive about their obesity they may be less likely to listen to and accept the risk messages given by them [26].

Using a qualitative design, our study sought to understand individuals' perceptions and interpretations of public health messages. Specifically, we aimed to expand on existing quantitative studies by exploring the ways in which obese individuals interact with public health messages about risk, how they understand and apply these messages to themselves and how their personal and social contexts and experiences may influence these perceptions and priorities.

## Methods

### Research Questions

We developed two broad questions to guide our study:

1) How do individuals interact with public health messages about the health risks of obesity?

2) What influences the ways in which individuals interpret these messages?

### Approach

The information presented in this paper was part of a broader qualitative study 'Obesity Have Your Say', which aimed to explore the health and social experiences of obese Australians [27]. This section of the study asked individuals to reflect on their opinions and understandings of public health messages about obesity, and explored the factors that influenced and shaped these opinions. We used four key open ended questions to explore these perceptions:

1) *What do you think about the range of public health messages that are currently around about obesity? What do you think is the key message they are giving?*

2) *Do you think the messages in these campaigns apply to you? How so? What about the general population?*

3) *Do these types of messages impact on your feelings about your weight? Why?*

4) *Do you think public health messages about obesity need to be changed? Why?*

We gained ethics approval from the Monash University Standing Research Ethics Committee.

### Sampling and Recruitment

Qualitative studies have been classified by some on a hierarchy of evidence ranging from single case studies through to generalisable studies [28]. To ensure the representativeness of this sample, we employed a diverse range of purposive, theoretical and strategic sampling methods [29,30] to recruit participants who were representative of a broad range of socio-cultural, demographic, geographic and weight groups within the obesity range, allowing us to explore the findings in the context of socio-cultural risk communication literature. We aimed to include individuals who were actively trying to lose weight or improve their overall health and wellbeing, those who were happy with their weight and those who had 'given up' trying to address their weight.

Methods used to recruit participants included local media (including radio, newspaper, television, and magazine articles), Internet advertisements (including electronic advertisements, mailing lists, a study website, and postings on Internet newsletters and forums), direct recruitment (through health professionals and personal trainers), advertisements in obesity and weight loss centres (such as Jenny Craig and Overeaters Anonymous), posters and flyers left in community areas (including shopping centres and local gyms), and workplace, university and hospital mailing lists and newsletters. Each of these recruitment strategies were used in a step by step approach to target different types of individuals. For example local media recruited a broad cross section of the community, postings on websites targeted individuals in the Fat Acceptance community, weight loss centres were used to actively recruit individuals trying to lose weight, and local gyms to recruit individuals who were engaging in exercise. These flyers were headed "Obesity. Have your say!" and called for obese individuals to discuss their experiences with their weight, their attitudes towards current public health messages about obesity, and societal attitudes towards obesity. An 1800 free-call number was provided, as was an email address and website. Additional detail about the sampling strategy can be found in Thomas et al. [31].

### Data Collection

A semi-structured interview schedule was designed. In depth semi structured telephone interviews were conducted between April 2008 and March 2009. Interviews lasted between 60 and 90 minutes, were audio-taped with participants' permission and transcribed by a professional transcribing service within seven days of being conducted.

### Data Analysis

Data analysis was conducted by SL and ST. Our analysis was guided by Grounded Theory techniques [32], whereby we read and re-read transcripts, coded, and identified categories/themes (and similarities and differences between these), sorted data to ensure that the concepts/theories were appropriate and noted differences between different groups of individuals [32,33]. Regular meetings were held to interpret and discuss findings, to build thematic areas and theoretical concepts, and to identify and refine new research questions and directions as they emerged from the data.

### Presentation of Data

Throughout this paper we use quotes to illustrate the research findings, with additional quotes for further reading presented in Additional file 1, Table S1. Because of the large numbers of participants in this study we have also quantified some of the responses. This allows us to identify the proportions and types of individuals who responded in certain ways. Where we have not used numbers, we have used the terms 'a few' to refer to less than a quarter of participants; 'some' to refer to 25-50% of participants; 'many' to refer to 50-75% of participants; and 'most' to refer to over 75% of participants.

## Results

### Overview

Of the 172 individuals who enquired about this study, 142 participated. 22 were excluded from the study as their BMI was less than 30, or they lived outside of Australia. A further eight refused participation due to the length of the interview or because they thought that the study was testing a new weight loss solution. Table 1 outlines the participant characteristics. Most were female (n = 106, 74.6%), with an average age of 44.8 years (19-75 years), married or in a de facto relationship (n = 92, 64.8%), and with a university degree (n = 89, 62.7%). Most participants were from middle (n = 59, 41.5%) to upper (n = 33, 23.2%) socio-economic income groupings and self identified as Australian born (n = 103, 72.5%). Three quarters of participants (n = 107, 75.4%) reported at least one physical health problem, and about a quarter of our participants (n = 40, 28.2%) reported that they had a mental or emotional health problem.

**Table 1:** Participant Demographics

Demographic category	n = 142 (%)
**Gender**	
Female	106(74.6%)
Male	36(25.4%)
**Age**	
Mean	44.8
Range	19-75
BMI (n = 141)*	
Mean	39.3
Range	30.0-71.7
30-39.9	88(62.0%)
≥ 40	54(38.0%)
**Marital status**	
Single	50(35.2%)
Married/De facto	92(64.8%)
**Country of Birth**	
Australian born	103(72.5%)
Born outside Australia	26(18.3%)
Not revealed	13(9.2%)
**Education**	
< High school	20(14.1%)
High school graduate < university degree	33(22.2%)
University or postgraduate degree	89(62.7%)
**Income before tax (AUD)**	
<50,000	48(33.8%)
50,000-100,000	59(41.5%)
>100,000	33(23.2%)
Not revealed	2(1.4%)

*One participant did not reveal height or weight to calculate their BMI

Because of the complexity of the data, this paper presents an overview of the key emerging themes for the group as a whole. We do however provide some commentary within the paper when results indicated very clear differences between some groups of participants. We have also provided descriptors of the gender and Body Mass Index (BMI) for each narrative used within the text.

### Perceptions of how public health campaigns apply to the general population

Participants' narratives were complex and at times contradictory. At the most basic level, the vast majority agreed with the basic risk concepts promoted in public health messages - that obesity was a risk factor for poor health outcomes in the general population (n = 117; 82%):

*"In the long term you are looking at diabetes, you are looking at joint replacements, you're looking at heart failure, you're looking at strokes, you're looking at so many negative health issues as a result of severe weight gain." *(57 year old female, BMI 33.4)

However, when we explored these opinions in more detail most female participants believed obesity was a minor risk factor for poor physical health outcomes but a major risk factor for poor mental health outcomes. Yet issues about the mental health consequences of obesity are very rarely mentioned in public health campaigns. Individuals believed mental health risk was caused more by societal reactions to obesity, rather than obesity itself:

*"It's mainly because of the way other people perceive fat people and the way other people treat fat people." *(34 year old female, BMI 46.1)

Many participants felt that public health based messages were misplaced by focusing too much on the physical health risks associated with *"being fat" *and did little to resonate with the overall health experiences of individuals who were very overweight. Some, particularly those who had given up trying to lose weight, also felt that there was too much emphasis on weight loss and weight measurement as a preventive message, with little emphasis on strategies to help those who found it difficult to manage obesity and the consequences there of:

"[messages should] *be more aimed at the day to day stuff - how to decrease joint pain, how to decrease shortness of breath, how to decrease having puffy feet every day." *(42 year old female, BMI 56.4)

Some felt that public health messages were disempowering for individuals because they only provided a small part of the overall message. By placing an overwhelming focus on the consequences of being overweight, participants felt that messages did little to convey the complex range of causes associated with obesity and the comprehensive solutions needed to help those who were very overweight. Participants felt that the absence of these two parts of the overall message pushed individuals towards radical, *"quick fix"*, unproven, high cost solutions, which were often ineffective in helping individuals to improve their health and wellbeing. Many male participants spoke about the lack of 'solution based' messages for individuals:

*"I think that while it's good that they can say go out there, lose weight, be healthier, these are the dangers of being overweight, I don't remember them ever saying 'this is where you can go where this has worked for people in the past', 'this is proven', 'stay off these pills and radical crap weight loss things'. The information's out there, but I don't see the support." *(27 year old male, BMI 32.5)

Many participants, in particular those with a BMI over 40, also felt that the focus on the health consequences of 'being fat' and the accompanying messages of personal responsibility amplified the "*guilt*", "*blame" *and "*shame*" that people experienced. Many felt that the public gaze was too firmly on the societal costs of individuals who had "*failed*" to maintain a "*normal weight*" rather than ways to help and support people:

*"Obesity is costing the government more money. You are doing a disservice to your government. It's your personal responsibility. It's all a result of your fatness. I look at it and go oh my god, what is happening here?" *(42 year old female, BMI 56.4)

Participants who stated that they were 'happy' with their weight completely rejected the public health messages about obesity. The most common example used by participants to disprove public health messages was their belief that whilst the population was getting 'fatter' they were also living longer. Others mistrusted the motives behind public health messages, stating that they had exaggerated the role of obesity in certain health problems:

*"I think all the messages about how terrible it is for your health have given people fears beyond what is real and reasonable. I know people who are actually fine, but they are totally scared they are going to drop dead of a heart attack because they are a bit fat. Scare tactics can be more damaging than any good." *(43 year old female, BMI 38.4)

Running through all narratives was the perception that public health messages unfairly focused too much attention on the outcome of the risk behaviour (obesity) in comparison with the risk behaviours that lead to obesity (i.e. unhealthy eating, sedentary lifestyle, excessive alcohol consumption, stress):

*"I think they need to stop putting all the emphasis on fat. I think that insisting that being thin is healthy is actually doing more harm than good. I think there needs to be more emphasis on eating well, exercising, looking after yourself, getting enough sleep at night which a lot of people don't, things like that rather than being thin. Those horrible 'Reduce Your Waist, Reduce your Risk' ads and stuff need to go away." *(22 year old female, BMI 45.9)

### Perceptions of how public health campaigns apply to themselves

Whilst the majority of participants believed that obesity had played a role in their poor physical health outcomes, over half felt that their obesity posed them only "*minor*" physical health risks (n = 74, 52%), by impacting on their "*mobility*" (n = 40, 28%), "*energy levels*" (n = 21, 15%) or "*fitness*" (n = 13, 9%). However, 82 women and 20 men spoke extensively about the impact of societal reactions to their weight (including the messages about obesity) on their emotional wellbeing. They felt the public glare on their weight "*constantly*" or "*all the time*". In particular, participants with a BMI over 40 stated that far from motivating them to "*lose weight*" they felt a "*failure*", questioned themselves: "*what's wrong with me?*" or felt "*frustration*" at not being able to achieve a "*societal norm*":

*"From my perspective they just highlight the fact that I'm a failure. There's a public campaign telling me that I need to have a campaign about me. People think that we want to be fat, people think that obese people are just uncontrollable gutses and choose to put themselves where they are. My choice in being obese is no more than somebody's choice of being homosexual." *(55 year old male, BMI 60.7)

Many of these participants described the long "*battles*" they had had with their weight - often since childhood, the constant processes of gaining and losing weight, and the complex reasons behind their overweight. Because of these personal experiences many participants stated that the social stereotypes of obesity and simplistic messages present in many public health campaigns made them feel even more "*isolated*", "*different*" and "*distressed*" when they were unable to meet the *"norms" *promoted by campaigns:

*"For a lot of people like myself losing weight isn't as simple as going for a jog every morning. For a lot of people their weight isn't any indicator of their health. So when you look at all these people who don't fit into the mould of 'eat right, exercise and you'll be thin and beautiful'; that's 90% of people who are seeing these campaigns are going to fall into this category." *(22 year old female, BMI 45.9)

Some individuals explained that obesity worked in combination with a number of different types of risk factors - such as smoking, drinking and inactivity - to contribute to poor health outcomes. As such, many felt that public health messages increased public assumptions that obesity was the sole cause of being unhealthy: *"If you're fat then you are automatically a health risk"*. Participants explained that different people would have different health outcomes depending on a complex range of factors, describing how some health conditions said to be caused by obesity also afflicted those who were "*thin*" or "*skinny*". Simplistic social marketing messages about 'eating less and exercising more' denied both the complexity of the causes of their obesity, and their abilities to act on these messages:

*"I think public authorities believe everybody should be thinner. There are obviously lots of people out there that are just really, really big. And I think if those people could have done anything about that, they would have done something by now. So just telling them 'ooh, you're too big, you should lose weight' - how has that helped anything?" *(39 year old female, BMI 43.7)

Many participants also stated that they could not relate to the behaviours of obese people, as depicted in these campaigns:

*"Whenever you go to your local doctor you see pictures of large, overweight men or large, overweight women eating hamburgers and thickshakes and saying 'one in three people over the age of 45 in this weight range are likely to suffer heart attacks'. I think, well that's not me, because I don't eat a hamburger and large chips every day." *(48 year old male, BMI 31.0)

Women (n = 26, 18%) spoke extensively about how health problems - ranging from Polycystic Ovarian Syndrome to depression - had led to their weight gain, and had prevented them from losing weight:

*"I have Chronic Fatigue Syndrome, I have a problem with the insulin sugar, I have low thyroid and I have adenomyosis. All of that causes me to maintain overweight and restricts me from doing physical activities." *(39 year old female, BMI 43.7)

Women diagnosed with mental health problems (n = 12) said that their emotional difficulties and the medications they were prescribed made them "*less inclined to be active*", or "*eat more*". Whilst most of these participants prioritised their mental wellbeing over their obesity "*if it's a choice between being fat or suicidal I'd rather be fat*", one participant had decided to stop taking her antidepressants and risk her mental wellbeing to be thin:

*"I want to seriously lose weight. I would rather be depressed and slimmer than fat and on drugs for depression." *(50 year old female, BMI 32.6)

These 12 women felt an acute sense of stigma and stereotyping from public health messages and felt that they had been unfairly labelled for their weight gain. Again, this suggested that the 'cause and effect' simplicity of some public health messages made individuals feel unfairly blamed for something that was beyond their personal control.

### Responses to risk: The 'responsible citizen'

Whether participants believed public health messages about obesity or not they were keen to point out that they were trying to maximise their health and wellbeing. Participants stated that they took a variety of health "*precautions*" including "*I lead a healthy lifestyle*", "*eating healthy*", "*I don't eat fatty foods*", "*I exercise*" and "*I'm active*". As such, participants were reframing the risk message away from 'obesity' and towards the 'risk behaviours' associated with poor health outcomes. Some stated that they made sure that they had "*regular health checks*" - thus portraying themselves as responsible and proactive in monitoring their health outcomes. Those with chronic health conditions such as arthritis, Type II diabetes and cardiovascular disease tried to minimise the impact that these conditions had on their life by stating that they were "*under control*", "*not a problem*", "*not very severe*", "*not too much of an issue" *or were only a small part of an otherwise healthy life. Some described that they were not a burden on the community, stating that they rarely or "*never needed to use medication*". A few participants with a BMI over 40 stated that whilst their weight had stopped them personally from doing things they enjoyed, it did not affect other people. In doing so, they tried to individualise the impact that their health problem had - stating that it affected them but not others:

*"My weight doesn't actually affect anyone else. It may affect what I do with other people, but it doesn't actually affect anyone else." *(29 year old female, BMI 50.4)

Participants under 50 years old rarely thought that their weight impacted on their health. However, these participants emphasised that if their weight did impact on their health they would take personal responsibility for addressing this:

*"I'm not really at that point where I need to lose the weight or I'm going to have some serious health effects tomorrow kind of thing. If it came to that I'd do it." *(44 year old female, BMI 32.0)

Again, this reinforced the personal responsibility frame of public health messages - that obese individuals are personally responsible for their own health and wellbeing and for changing their behaviour to enable weight loss.

### Reframing public health messages away from 'obesity' and 'weight loss'

The vast majority of participants wanted the focus of public health messages to shift "*away from weight and body mass index and focus on health*" and to emphasise the benefits of engaging in healthy lifestyle practices rather than the negative outcomes of 'risky' behaviour. In addition they wanted public health campaigns to link risk messages to practical solutions and support, such as ways that all individuals (no matter what their size) could increase the amount of incidental physical activity in their daily lives:

*"The message needs to be, you need to be fit whatever your weight. And that's why I say it needs to be health at every size." *(51 year old female, BMI 43.8)

They also felt that messages needed to strike a better balance between conveying the risks whilst also minimising possible stigmatisation of obese individuals - in effect by targeting obesity rather than obese individuals:

*"Most of the harm that comes to overweight people, most of what makes them feel so crappy, basically comes down to being told that they're not good enough and they're worthless. I think that there needs to be some effort put in to making overweight people realise that they are not big worthless lumps of nothing, that they are good people and sexy people and smart people. Because when you are fat you're treated like that's all you are and you're never going to amount to anything unless you can get rid of it. I think people are missing out on life and being happy because they're just being constantly told how horrible they are. So I think more than anything overweight people need a good boost of self esteem." *(22 year old female, BMI 45.9)

Finally they wanted to see campaigns that recognised that different groups could have different health outcomes and that therefore they could not be grouped *"in the same box".*

## Discussion

The results of this study reveal a great deal about the ways in which obese individuals interact with current public health messages about obesity. In addition, they provide useful pointers about how these messages could be reframed to make them more effective - particularly for those who are already significantly overweight.

### There is a perceived mismatch between messages and experiences

There appears to be a mismatch between public health messages about obesity and the experiences of obese individuals. Socio-cultural contexts, as well as individuals' own experiences with weight, strongly influence how individuals feel about public health messages. In particular, individuals feel that the messages are 1) too simplistic, 2) stereotyping, 3) overly focused on physical health risks, 4) fixated on 'fatness' rather than the lifestyle factors that may negatively influence health outcomes, and 5) not reinforced with broader information to educate and support people to make changes in their lifestyles. As in other studies, most individuals in this study understood and agreed with the basic 'health risk' messages behind public health campaigns [8,34,35]. However, they disagreed with the ways in which messages were framed, communicated and delivered. In particular they were concerned with the fact these messages suggested that individuals were not taking personal responsibility, made moral judgements about 'lazy' or 'irresponsible' behaviours, or implied that the problems could be fixed through simply 'eating less and moving more'.

### Messages are overly simplistic

Participants felt that the messages about obesity were overly simplistic and narrowly focused, ignoring both the ongoing struggles that many obese individuals have with their weight as well as the broader influences of the socio-cultural and physical environments [36]. Whilst public health messages seemed to imply that there is an easy solution and that individuals should be able to lose weight merely by exercising enough self-control, participants' own experience showed that there was in fact no easy solution. Public health messages which do not consider underlying social and cultural factors and which rely on the role of personal responsibility, may compound this problem, leading people to feel blamed and disconnected from the message [26]. Furthermore, the narrow focus of public health campaigns on weight loss rather than improvements in health may encourage individuals to adopt unsustainable weight loss diets or reinforce discrimination against them. In current usage the word 'obesity' is not a neutral term but it is laden with moral judgments and biases about people's characters. By overemphasising weight, the messages may generate poor mental health outcomes for obese individuals or discrimination [37].

### Messages are stigmatising

Overly simple messages about complex problems may not only be ineffective but can be stigmatising for those whose behaviour they are trying to change. Furthermore, as this study has found, it may cause them to disconnect from the message [26]. Stereotypical images depicting obese people as engaging in personal misbehaviours - e.g. eating junk food, eating too much or being sedentary - appeared to reinforce the impression that all obese people are 'unhealthy', 'high risk', 'morally deviant' and 'to blame' for their health outcomes. If people perceive that public health messages stigmatise them they may become more vulnerable to the risks [38,39]. For example, in HIV/AIDS, stigmatisation of gay men, drug users and sex workers made these people more vulnerable to infection and disconnected them from messages and interventions designed to reduce their risk [40].

In response to the sense of stigmatisation, many people in this study rejected the view that public health campaigns about obesity applied personally to them. They sought to deflect blame away from themselves by claiming that they were responsible, healthy, active and contributing members of society who followed public health guidelines - such as eating a healthy diet, engaging in regular physical exercise and having regular health checks [41]. Such assertions may reflect their reactions to constant messages that they are irresponsible and at 'high risk' for disease. Participants' perceptions of stigma highlight the difficulty faced by public health campaigns about obesity in developing an approach that provides useful information to the population as a whole without stigmatising, blaming or isolating the minority of individuals who are already obese. This is in fact not only a concern in the area of obesity but also in relation to the problems of health inequality, social disadvantage, and access of older people to health care, where those in need are often blamed for driving up costs [42]. Whilst all public health interventions should be evaluated to see if they cause or amplify stigma, it may be that in the area of obesity stigma is so socially engrained that we now need strong anti-stigma messages. Public health based anti-stigma interventions in relation to other health issues such as mental illness have been important in improving public understanding, health professional training, and encouraging individuals to seek help and support [43]. Given the highly adverse effects of obesity stigma on mental health and help seeking behaviour there would seem to be a strong case for public health agencies working in obesity to develop practical guidelines and strategies to address this issue.

### How might the messages be reframed?

Participants identified a need for change both in the key messages of public health campaigns and in the ways in which these messages are delivered. They wanted a marked shift away from negatively framed messages which make moral judgements about their 'risky behaviours' towards more positive messages about the benefits of healthy behaviours, particularly around exercise. Considering both the evidence that one can be 'fit and fat' and that weight loss programs rarely lead to sustained weight loss, public health campaigns should use outcome measures other than weight and body mass index [44].

Whilst general messages are important we need to ensure that these messages don't underplay the complexity of the causes of obesity. Public health messages aimed at influencing individuals may have limited success as they do not reflect the broader socio- cultural, economic and physical environments that affect individuals' health behaviours and outcomes [44-46]. Policy makers must also ensure, whenever adopting a universal message, that it does not inadvertently harm a particular group [26]. Different individuals and groups will value and respond to different types of messages in different ways. Messages that resonate with some may distance others. In addressing these concerns user perspectives and engagement should be commonplace in the development and design of public health messages, enabling individuals to design and shape the messages to suit their particular groups [45,47].

## Limitations

There were several limitations to this study. Firstly this was a self selecting sample who actively 'opted in' to this study. As such, the participants in this study may not be representative of the broader population. Secondly, the sample was skewed towards older women from higher socioeconomic and education groupings and did not specifically collect data about ethnicity. Finally, as with any qualitative study, the analysis of transcripts was based on the researchers interpretation of the meanings within the data. Whilst steps were taken to ensure that there was consistency in the interpretation of the data within the research team, the data may be interpreted differently by those with different disciplinary or theoretical approaches.

## Conclusions

This study provides important qualitative data about obese individuals' reactions to public health messages about obesity. The study shows that many personal and contextual factors influence the ways in which different types of individuals interpret and apply public health messages to their own health and wellbeing. It also shows that some individuals may find broad messages personally stigmatising and that this may be an unintended consequence of well-meaning public health strategies. Whilst the findings of this study may not be generalisable to all obese adults, it provides important understandings about how public health messages resonate with different groups of individuals. However, much more research - both qualitative and quantitative - is needed to continue to enhance our understanding of the impact of obesity messages on individuals.

## Competing interests

The authors declare that they have no competing interests.

## Authors' contributions

SL: Designed study, collected data, conducted interviews, conducted preliminary data analysis, drafted paper. ST: Study Chief Investigator, designed study, conducted data analysis and interpretation, contributed to drafting of paper. JH: Study Chief Investigator, designed study, contributed to data interpretation, contributed to drafting of paper. DC: Study Chief Investigator, designed study, contributed to data interpretation, contributed to drafting of paper. WB: Study Chief Investigator, designed study, contributed to data interpretation, contributed to drafting of paper. PK: Study Chief Investigator, designed study, provided critical feedback on draft of paper. All authors have read and approved the final manuscript.

## Pre-publication history

The pre-publication history for this paper can be accessed here:

http://www.biomedcentral.com/1471-2458/10/309/prepub

## Supplementary Material

Additional file 1 **Table S1 Additional quotes**. Additional quotes from participants to further illustrate the research findings. Click here for file
