# Supplementary material for: "I don't eat a hamburger and large chips every day!" A qualitative study of the impact of public health messages about obesity on obese adults
Source: BMC Public Health. 2010 Jun 4;10:309. doi: 10.1186/1471-2458-10-309 (PMC2887828; doi:10.1186/1471-2458-10-309)
Supplement: Additional file 1 — Table S1 Additional quotes. Additional quotes from participants to further illustrate the research findings. [file 1471-2458-10-309-S1.DOC]

**Additional file 1, Table S1 – Additional Quotes**

**Perceptions of how public health campaigns apply to the general population**

*“The negative mental health of being obese, I keep thinking it’s not the obesity...it’s the response that other people have to you and what they assume about* [you]*, like people will assume that I don’t do any physical activity, people assume that I’m not fit...there’s all these subtle messages about not being feminine, not being physically active, not being acceptable, and you’d have days like that sometimes where you’d just want to crawl under a rock somewhere. It’s hardly surprising that people with weight problems – living with that sort of degree of prejudice in our society must contribute to their feelings of poor health.”* (51 year old female, BMI 43.8)

*“There is no encouragement. The bottom line* [of public campaigns] *is you are going to get cancer and die [laughs]. That is why I reckon they are not working. If I am going to die, I might as well die happy and fat.”* (61 year old male, BMI 30.9)

*“It's disempowering to people to be constantly told all the time from above, from this authority, you're going to die, you're going to do this, you're going to put a strain on Medicare, you're going to get cancer and heart disease and all of this. I think it’s shameful. Shame never led anyone to take action. It means you want to hide yourself away in a room.”* (32 year old female, BMI 38.4)

*“I don’t buy into the whole people with obesity are pushing up our health costs garbage. I think you will find that much of what is reported as being an estimated health cost of obesity is actually a lot of this movement and light and dietary clap trap.”* (37 year old female, BMI 46.2)

*“I think they overall have a negative impact. I think that they stigmatise obese people by making a special thing of them. I think that there’s some debate about whether obesity is the cause of negative health outcomes or a symptom. I also think it is quite possible to be fit, healthy and obese.”* (44 year old male, BMI 43.4)

**Perceptions of how public health campaigns apply to themselves**

*“I always feel like I’m a real loser, I get angry with myself for not being able to lose weight. I feel guilty that I don’t lose weight, I feel ashamed. Yes it has a big affect on my psychological wellbeing.”* (62 year old female, BMI 38.7)

*“Last night I played one to one basketball with my 20 year old nephew and could only do it so much because it was exhausting. I wouldn’t solely attribute that to weight but a range of factors, the fact that I smoke, the fact that I’m overweight, the fact that I’m 40 and he’s 20. I wouldn’t like to assume that purely one element of my physicality is completely dominating my inabilities to do things.”* (42 year old male, BMI 35.8)

*“It’s just a...label that’s put on anybody that’s got any sort of weight problem...As soon as you do put any sort of weight on...it doesn’t matter who it is, they instinctively turn around and they automatically just say well you are going to suffer with diabetes and you are going to have heart troubles, and you are going to have cholesterol problems, and if you don’t fix it now you're going to be dead within 6 months type thing.”* (48 year old female, BMI 59.4)

*“Just because you’re thin doesn’t mean you don’t have high cholesterol or high blood pressure”* (29 year old female, BMI 39.8)

*“I was told by my doctor that the average woman with polycystic ovarian syndrome who eats a normal diet that everyone else eats ...will put on between 6 and 12 kilos a year.”* (29 year old female, BMI 50.4)

**Responses to risk: The ‘responsible citizen’**

*“My heart’s pretty healthy I’ve had a stress test and my results were very good. They said your heart’s got a great response to exercise and stress and that there’s no problems there.”* (51 year old male, BMI 40.8)

“*Everything else is good, everything else about me’s healthy; I don’t suffer anything else.*” (43 year old female, BMI 37.5)

**Reframing public health messages away from ‘obesity’ and ‘weight loss**

*“‘The benefits add up’ is the message that people need to get so it doesn’t really matter where you start so long as you start.”* (39 year old male, BMI 31.8)

*“Take the focus off weight and just put the focus onto health. You can be a larger person with absolutely no health issues and you can be a skinny person with phenomenal health issues. So instead of targeting your body size it should be targeting just general health overall like targeting blood pressure or targeting sugar levels rather than targeting body size because it’s really unrealistic to assume that all overweight people are sick and all skinny people are healthy and that’s really the way it’s focused at the moment. So if it’s targeted back onto how well are you feeling not how big is your dress size that will probably be one of the major steps.”* (40 year old female, BMI 57.3)

*“Anyone who wants to do good in the world and wants to help fat people should be putting in the message to be active, eat well and stop obsessing about it. You know it is so psychologically unhealthy, the model of you know, hoard people off into getting surgery and doing stupidly intense dieting and the whole competitive weight loss on television.”* (43 year old female, BMI 38.4)

*“Things that just say if you’re fat then you automatically have a health risk, when I read that sort of simplistic stuff I say well they don’t know what they’re talking about.”* (51 year old male, BMI 36.8)
